# Morin Attenuates *Streptococcus suis* Pathogenicity in Mice by Neutralizing Suilysin Activity

**DOI:** 10.3389/fmicb.2017.00460

**Published:** 2017-03-20

**Authors:** Gen Li, Gejin Lu, Zhimin Qi, Hongen Li, Lin Wang, Yanhui Wang, Bowen Liu, Xiaodi Niu, Xuming Deng, Jianfeng Wang

**Affiliations:** ^1^The First Hospital and Institute of Infection and Immunity, Jilin UniversityChangchun, China; ^2^Key Laboratory of Zoonosis, Ministry of Education, Institute of Zoonosis, College of Veterinary Medicine, Jilin UniversityChangchun, China

**Keywords:** *Streptococcus suis*, suilysin, morin, anti-infective, molecular modeling

## Abstract

*Streptococcus suis*, a Gram-positive pathogen, is widely recognized as an important agent of swine infection, and it is also known to cause a variety of zoonoses, such as meningitis, polyarthritis and pneumonia. Suilysin (SLY), an extracellular pore-forming toxin that belongs to the cholesterol-dependent cytolysin family, is an essential virulence factor of *S. suis* capsular type 2 (SS2). Here, we found that morin hydrate (morin), a natural flavonoid that lacks anti-SS2 activity, inhibits the hemolytic activity of SLY, protects J774 cells from SS2-induced injury and protects mice from SS2 infection. Further, by molecular modeling and mutational analysis, we found that morin binds to the “stem” domain 2 in SLY and hinders its transformation from the monomer form to the oligomer form, which causes the loss of SLY activity. Our study demonstrates that morin hinders the cell lysis activity of SLY through a novel mechanism of interrupting the heptamer formation. These findings may lead to the development of promising therapeutic candidates for the treatment of SS2 infections.

## Introduction

*Streptococcus suis* (*S. suis*) is a Gram-positive coccus and emerging zoonotic agent whose global spread could cause a variety of life-threatening infections, including, but not limited to, sepsis, arthritis, meningitis, encephalitis, endocarditis and pneumonia in swine and humans (Lun et al., 2007; Goyette-Desjardins et al., 2014). So far, there are 35 (1–34 and 1/2) official serotypes that have been identified based on capsular antigens (Gottschalk et al., 2007). Serotype 2 is the most frequently isolated from clinically diseased piglets (Yang et al., 2006; Aspiroz et al., 2009; Okura et al., 2016) and is responsible for significant economic losses to the porcine industry worldwide, causing more than $300 million in losses in the US alone (Heidt et al., 2005). Since 1968 when the first human case of *S. suis* infection was reported in Denmark, more than 1600 human cases have been documented worldwide (Hedegaard et al., 2013). Most of these infections occur in people who work with pigs or in those who are in close contact with raw pork products in Southeast Asia, where there is a high density of pigs (Wertheim et al., 2009). In China, two large outbreaks of *S. suis* infection were recorded in 1998 and 2005, which caused substantial economic losses to the pig industry and many human deaths, representing a significant public health concern (Ye et al., 2006; Yu et al., 2006).

To successfully establish an infection, *S. suis* must overcome epithelial barriers, evade immune attack, survive in the bloodstream and invade various organs, deliberately causing exaggerated local inflammation. Each of these steps is mediated by multiple stages of virulence factors synthesized by the bacterium (Fittipaldi et al., 2012). Therefore, the targeting of such virulence determinants may be a promising therapeutic strategy for the treatment of *S. suis* infections. Suilysin (SLY, encoded by the gene *sly*), a 54 kDa extracellular pore-forming protein that belongs to the family of cholesterol-dependent cytolysin, is a virulence-associated factor secreted by most virulent *S. suis* strains and is regarded as a virulence marker of *S. suis* (Tenenbaum et al., 2016). Over the past 20 years, SLY has been shown to lyse different cell types, which would affect complement activity, increase the blood-brain barrier and/or blood-cerebrospinal fluid barrier permeability, induce the inflammatory reactions of host and facilitate bacterial infection (Du et al., 2013; Leung et al., 2014). Staats’s group reported that 5 of 19 strains of capsular type 2 *S. suis* are SLY positive, which are highly virulent in pigs based on mortality, morbidity and pathology. While the remaining 14 SLY-negative strains are lower virulent or avirulent (Staats et al., 1998). In a recent study, Takeuchi et al. generated a*sly*-knockout strain and identified SLY as an important virulence factor in a mouse model of *S. suis* infection (Takeuchi et al., 2014). Approximately 90% of mice inoculated with *S. suis* ST1, a strain that produces high levels of SLY, were dead within 10 days. In contrast, similar infections with *sly*-knockout ST1 or the *S. suis* ST104 strain, a natural isolate that produces only low levels of SLY, failed to cause mortality within the same experimental timeframe. Furthermore, their study illustrates that the *S. suis* strain with higher-level production of SLY more frequently causes meningitis. In summary, SLY was confirmed to contribute to higher bacterial density, enhanced inflammation in the brain, increased mortality in mice and the pathogenesis of meningitis in humans (Takeuchi et al., 2014). Therefore, SLY may represent a novel therapeutic target for the treatment of *S. suis* infection.

Morin hydrate (morin), a yellow natural bioflavonoid compound found in many members of the Moraceae family and various fruits and vegetables, has been shown to possess multiple pharmacological effects, including anti-inflammatory, anti-proliferative and anti-oxidant activities (Krol et al., 2002; Fang et al., 2005; Green et al., 2012; Lee et al., 2016). Additionally, several recent studies have shown that morin can significantly attenuate the virulence of pathogenic bacteria (Huang et al., 2014; Wang et al., 2015; Sivaranjani et al., 2016). However, to our knowledge, the influence of morin on SLY and *S. suis* virulence has not been reported previously. In this study, morin was identified as an effective SLY inhibitor by a hemolysis assay. Furthermore, the virulence of *S. suis* was significantly attenuated in the presence of morin. These findings suggest that morin could be a promising therapeutic candidate for the treatment of *S. suis* infection.

## Materials and Methods

### Bacterial Strain, Culture Conditions, and Morin Preparation

The bacterial strain ZY05719 (highly virulent *S. suis* serotype 2) used in this study was a gift kindly provided by Professor Hongjie Fan (Key Lab of Animal Bacteriology, Ministry of Agriculture, Nanjing Agricultural University, Nanjing, China). SS2 strain ZY05719 was cultured in Todd-Hewitt broth (THB; Qingdao Hope Biol-Technology Co., Ltd, Qingdao, China) at 37°C. Morin was commercially obtained from Chengdu Herbpurify CO., LTD (Chengdu, China) and was dissolved in dimethyl sulfoxide (DMSO; Sigma-Aldrich, St Louis, MO, USA) to make a stock solution (40.96 mg/ml).

### Production of Recombinant SLY Protein and Anti-SLY Rabbit Serum

*Streptococcus suis sly* cDNA was subcloned into pET-28a(+) vector (Novagen, Madison, WI, USA) with BamHI and NdeI restriction enzyme cutting sites to construct the prokaryotic expression plasmid pET-28a(+)-SLY. The recombinant plasmid encoding the histidine-tagged SLY protein (rSLY) was transformed in *E. coli* BL21. Recombinant protein expression was induced by the addition of 0.2 mM isopropyl-β-D-thiogalactopyranoside (IPTG) at 16°C for 16 h. The supernatant of bacterial cell lysates was loaded onto a Ni-NTA column to purify the resultant rSLY protein.

The expression vectors for the SLY-Tyr54Ala, SLY-Gln107Ala and SLY-Asp111Ala mutants were constructed using the QuikChange site-directed mutagenesis kit (Stratagene, La Jolla, CA, USA) based on pET-28a(+)-SLY and were verified by DNA sequencing. The expression and purification of these mutants was performed as described above for wildtype SLY (WT-SLY). The primers used in this study for the construction of pET-28a(+)-SLY and its derivatives are listed in **Table 1**.

**Table 1:** The binding free energy (kcal/mol) of WT-morin, Tyr54Ala-morin, Gln107Ala-morin, and Asp111Ala-morin based on a computational method and the values of the binding constants (*K_A_*) based on fluorescence spectroscopy quenching.

	WT-SLY	Tyr54Ala	Gln107Ala	Asp111Ala
Computational Method	–12.7 ± 1.9	–8.3 ± 1.7	–7.1 ± 1.3	–6.9 ± 1.1
*K*_A_ (1 × 10^4^) L⋅mol^-1^	7.2 ± 1.4	5.1 ± 1.5	4.9 ± 1.2	4.8 ± 1.2

Approximately 3 mg rSLY protein was sent to Tianjin Sungene Biotech Co., Ltd. (Tianjin, China) for the production of anti-SLY rabbit serum, which was confirmed by western blot to detect recombinant SLY protein and secreted SLY in bacterial culture supernatants.

### Anti-bacterial Activity of Morin against *S. suis*

SS2 was cultured in THB and diluted at a density of 5 × 10^5^ CFUs/ml. The minimal inhibitory concentration (MIC) of morin for SS2 was measured by a serial dilution method, according to the procedures of the CLSI guideline M31-A2 (CLSI, 2002). Lag-phase bacterial cultures were divided into 10 mL aliquots, and morin was added to yield the desired final concentrations of 0, 4, 8, 16, and 32 μg/mL. To determine the growth kinetics, bacterial densities were monitored every 30 min at an OD of 600 nm for 6 h.

### Hemolysis Assay

*Streptococcus suis* strain SS2 from an overnight culture was transferred (1:50) to THB with various concentrations of morin (0, 4, 8, 16, and 32 μg/mL) and further cultured to the stationary phase (OD_600_ = 2.5). Then, the bacteria were pelleted by centrifugation (10,000 rpm, 1 min) to obtain culture supernatants. Defibrinated rabbit blood was suspended in PBS to 2.5% vol at a final volume of 1 mL and was incubated with 100 μL culture supernatant at 37°C for 30 min in parallel with controls containing either no culture supernatants (negative control) or 2.5% vol Triton X-100 (positive control). After incubation, the reaction system was centrifuged (10,000 rpm, 1 min), and the optical density at 543 nm (OD543) of the supernatant for each sample was determined by ultraviolet spectrophotometry as previously described (Wang et al., 2015).

The effect of morin on hemolysis induced by rSLY or its derivatives was measured by incubating 100 μL purified proteins (100 ng/mL) with the indicated concentrations of morin in 1 mL PBS for 30 min at 37°C prior to the addition of 25 μL defibrinated rabbit blood. Cell lysis was determined as described above.

### Western Blot Assay

A total of 20 μL culture supernatants (as described above) were mixed with Laemmli SDS sample buffer, boiled for 5 min and separated by 12% sodium dodecyl sulfate polyacrylamide gel electrophoresis (SDS-PAGE). The protein was transferred to polyvinylidene difluoride membranes (PVDF, Bio-Rad, Hercules, CA, USA) and SLY was visualized by primary anti-SLY rabbit serum diluted 1:2000 and secondary horseradish peroxidase-conjugated anti-rabbit antiserum (Sigma–Aldrich) diluted 1:4000 with Amersham ECL Western blotting detection reagents (GE Healthcare, Buckinghamshire, UK), as described previously (Wang et al., 2015).

### Cell Culture and Infection

Mouse J774 macrophage-like cells were cultured at 37°C in a 5% CO_2_ atmosphere in complete medium (Dulbecco modified Eagle medium (DMEM, Invitrogen) supplemented with 10% fetal bovine serum (FBS), 100 U/mL penicillin and 100 μg/mL streptomycin). The cells were seeded at a density of 2 × 10^4^ cells per well in 96-well plates overnight and were subsequently infected with SS2 at the mid-log phase (OD_600_ = 0.8–1) of bacterial culture and resuspended in FBS-free DMEM medium at a multiplicity of infection (MOI) of 10 with various concentrations of morin for 5 h. Cells treated with 2.5% vol Triton X-100 or DMEM served as the positive and negative controls, respectively. The supernatants were harvested from each well by centrifugation (1,000 rpm, 10 min), and the presence of LDH released into supernatants was measured using the Cytotoxicity Detection Kit (LDH; Roche, Basel, Switzerland) according to the manufacturer’s instructions. Microscopic images of stained cells were further obtained under a confocal laser scanning microscope (Nikon, Tokyo, Japan) using live/dead (green/red) reagent (Invitrogen).

For the transwell experiment, 1 mL J774 cells (5 × 10^4^ cells/mL) in complete medium were plated in the upper chamber of 24-well transwell plates (Costar, 3.0 μm in pore size) for 3 days, and 500 μL complete medium without antibiotics was added in the lower chamber. At days 4, 6, and 7, the concentrations of FBS in the upper chamber were changed to 5, 1, and 0%, respectively. Upon reaching confluence after 8 days, the cells in each chamber were infected with 200 μL SS2 (1 × 10^8^ CFU/mL) at the mid-log phase (OD_600_ = 0.8–1) of bacterial culture and resuspended in FBS-free DMEM medium in the presence of 32 μg/mL morin for 15 min. The number of bacteria in the lower chamber was calculated by titrating on THB plates to determine the penetration ability of SS2 through J774 in the presence or absence of morin. The percentage of penetration = 100 × lower chamber CFUs/ upper chamber added CFUs.

### Mouse Model of *S. suis* Infection

Eight-week-old female C57BL/6J mice were purchased from the Experimental Animal Center of Jilin University (Changchun, China). The animal experiments were reviewed and approved by the Animal Care and Use Committee of Jilin University.

Overnight cultures of SS2 were transferred into THB (diluted 1:500) and grown at 37°C to mid-log phase (OD_600_ = 0.8-1). Cells were centrifuged (3,000 rpm, 10 min), washed once with PBS and suspended in PBS to a final concentration of 1 × 10^9^ CFU/mL. Mice were inoculated intravenously with 100 μL of the bacterial suspension. Two hours after inoculation, morin was administered subcutaneously at 100 mg/kg and at the same dosage at 8-hour intervals. Infected mice administered with DMSO served as the control group. The infected mice (30 per group) were observed for survival analysis for up to 4 days.

### Molecular Modeling

The structure of SLY was taken from the X-ray crystal structure in the Protein Data Bank (PDB) with PDB codes of 3HVN. The free protein obtained from the PDB (3HVN) was first equilibrated by a 100 ns molecular simulation on the solute, which was used for the molecular docking with morin. Then, the geometry of morin was optimized at the B3LYP/6-31G^∗^ level by the Gaussian 03 program. Subsequently, the standard docking procedure for SLY with morin was performed by using AutoDock4. The detailed process is reported in our previous paper (Dong et al., 2013; Wang et al., 2015).

In this study, complex systems were investigated by MD simulations using the Gromacs 4.5.2 software package based on the Amber99sb force field and TIP3P water model. The electrostatic term was described by using the particle mesh Ewald algorithm method, and all bond lengths were constrained by the LINCS algorithm. The initial velocity was obtained from a Maxwellian distribution. The desired initial temperature was 300 K. The density of the system was adjusted by NPT condition (*P_0_* = 1 bar, coupling time *τ_p_* = 0.5 ps). The protein and non-protein systems were coupled separately to the temperature bath. The antechamber programs and AM1-BCC partial atomic charges from the Amber 10 software were used to estimate morin. In this work, the binding free energies between SLY and morin were calculated by the Molecular Mechanis/Poisson-Boltzman Surface Area (MM-PBSA) method supplied with the Amber 10 package. Then, the interaction between inhibitors and each residue in the binding site of SLY was analyzed using the MM-PBSA decomposition process.

To verify the result of MD simulations, the interactions between morin and WT-SLY, SLY-Tyr54Ala, SLY-Gln107Ala, and SLY-Asp111Ala were investigated by fluorescence quenching. The proteins were used as the fluorophore and morin was used as the quencher. Commonly, fluorescence quenching can be described by the following Scatchard equation: *r/D_f_* = *nK*–*rK*, where *r* is the ligand amount of substance per mole of protein binding (*r* ≈*ΔF*/*F_0_*), *D_f_* is the free concentration of morin, *K* is the binding constant and *n* is the number of binding sites. In this system, due to the morin concentration being far greater than the concentration of protein; the *D_f_* is replaced by the total morin concentration. According to experimental results, the linear fitting plots of *r/D_f_ vs r* between morin and WT-SLY, SLY-Tyr54Ala, SLY-Gln107Ala, or SLY-Asp111Ala could be made. The values of *K* and *n* could be obtained based on the plots.

### Statistical Analysis

All experimental data (*n* ≥ 3) are expressed as the mean ± SD. GraphPad Prism 5.0 was used for statistical analysis using Student’s *t*-test. Significance levels of *P* < 0.05 and *P* < 0.01 are indicated in the figures.

## Results

### Morin Inhibits SLY Hemolytic Activity

**FIGURE 1:**
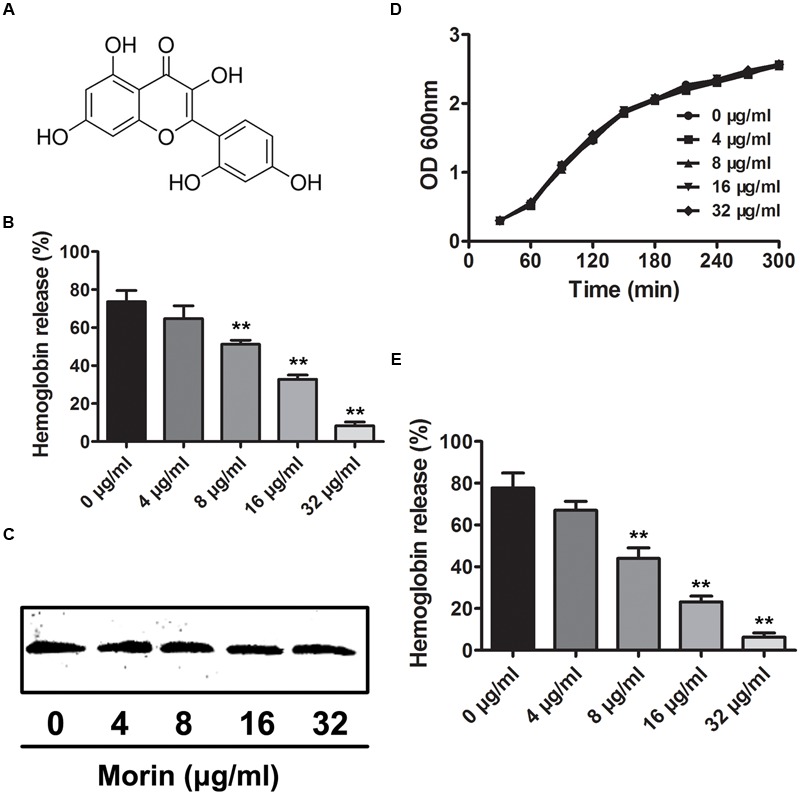
**Inhibition of SLY-induced hemolysis by morin. (A)** The chemical structure of morin. **(B)** The inhibitor effect of morin on the hemolytic activity of bacterial culture supernatants after co-culture with SS2. SS2 was co-cultured with morin, and the hemolytic activity of culture supernatants was determined using a hemolysis assay. The percent hemolysis of each sample was calculated by comparison with the control sample. **(C)** Western blot analysis of the SLY level in the culture supernatants. **(D)** The growth of SS2 in the presence of the indicated morin concentrations. SS2 was co-cultured with various concentrations of morin, and the growth kinetics were determined by measuring the OD600 of each sample every 30 min. **(E)** Inhibition of purified rSLY activity by morin. Following an incubation with the indicated concentrations of morin, the hemolytic activity of rSLY was examined by hemolysis assay. ^∗^ indicates *P* < 0.05, and ^∗∗^ indicates *P* < 0.01 compared with the morin-free sample (two-tailed Student’s *t*-test).

The hemolytic activity of culture supernatants from the stationary phase of SS2 was measured by a hemolysis assay in the presence of various concentrations of morin (**Figures 1A,B**). As shown in **Figure 1B**, the SLY protein secreted by *S. suis* at the stationary phase could sufficiently induce hemolysis, as 85.38% of red blood cells were lysed by the control sample in the absence of morin. However, upon co-culture with morin, the hemolytic activities of bacterial supernatants were significantly decreased in a concentration-dependent manner (**Figure 1B**). This finding suggests that such inhibition was due to a suppression of SLY activity by morin or a direct neutralization of SLY-mediated hemolysis by morin.

Western blot analysis was performed to detect the level of secreted SLY protein in culture supernatants and, interestingly, no visible difference was observed between control and morin-treated samples (**Figure 1C**). Additionally, the MIC of morin against SS2 was greater than 1,024 μg/mL, and morin had no influence on SS2 growth at the concentrations required to inhibit the hemolytic activity of culture supernatants (**Figure 1D**). These data indicate that morin has effectively no antimicrobial activity against SS2. Furthermore, the effect of morin on purified recombinant SLY (rSLY)-mediated hemolysis was evaluated, and, consistent with the results described above, the addition of morin significantly reduced the hemolytic activity of rSLY (**Figure 1E**). Taken together, these findings reveal that the inhibition of the hemolytic activity of bacterial culture supernatants by morin was not due to a decreased production of SLY in the supernatants but may be due to the direct binding of morin to SLY to neutralize SLY activity.

### Morin Alleviates SS2-Mediated Cell Injury

**FIGURE 2:**
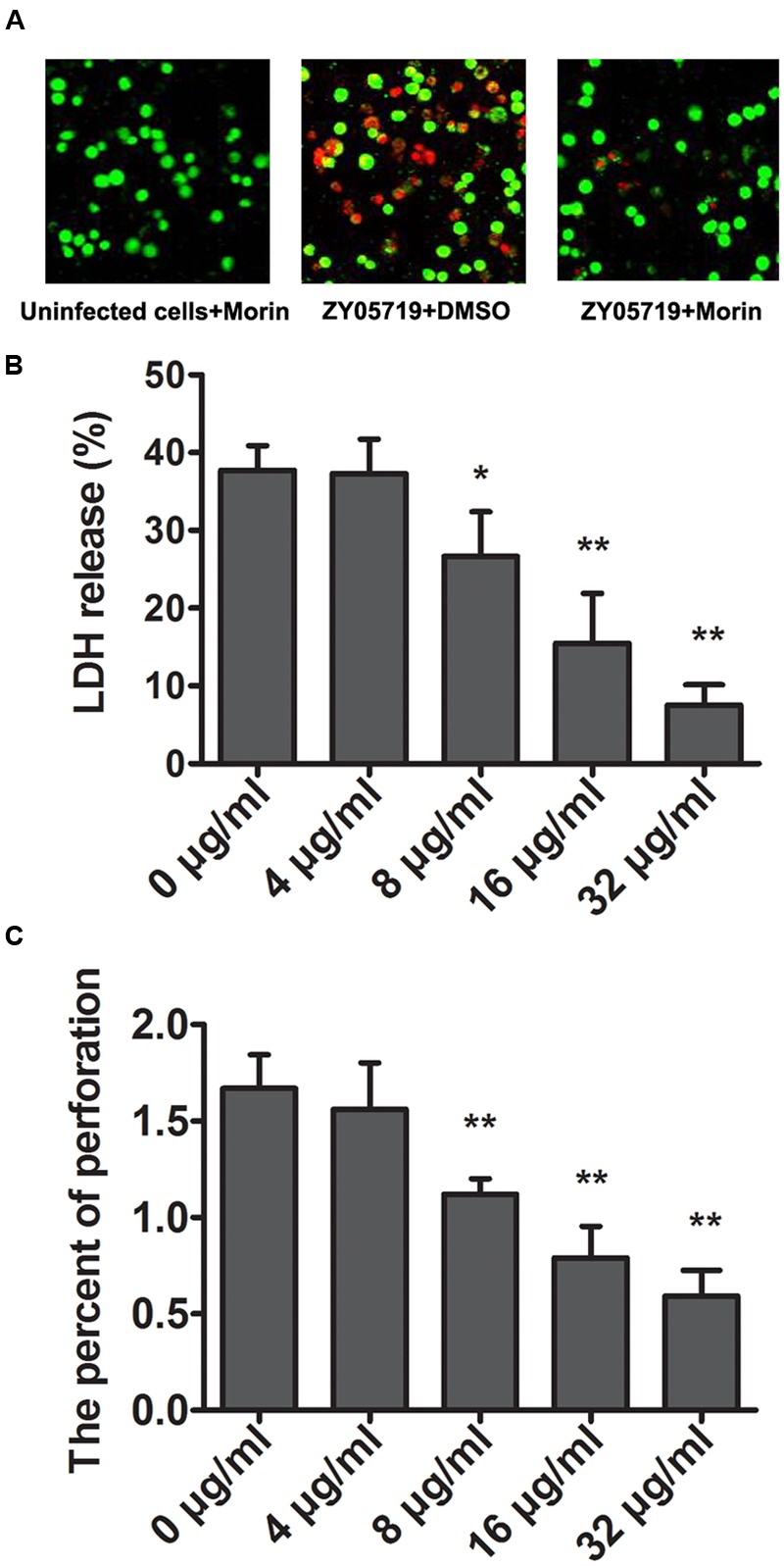
**Attenuation of SS2-induced cell injury and epithelial barrier penetration by morin. (A)** J774 cells were infected with SS2 in the presence or absence of morin, stained with live (green)/dead (red) agent and captured using a confocal laser scanning microscope. **(B)** The LDH level in the co-culture of SS2 and J774 cells treated with increasing concentrations of morin was determined using a Cytotoxicity Detection Kit (LDH) to quantify SS2-induced cell injury. Relative LDH release of each sample = (OD of tested group – OD of negative group)/(OD of positive group – OD of negative group) × 100%. **(C)** J774 cells were plated and cultured onto an epithelial barrier and then infected with SS2. The penetration rate of SS2 was examined by a CFU assay. ^∗^ indicates *P* < 0.05, and ^∗∗^ indicates *P* < 0.01 compared with the morin-free sample (two-tailed Student’s *t*-test).

A live/dead (green/red) assay and LDH assay were further employed to verify the potential protection of morin against SS2 *in vitro*. 37.73% cells were dead in the sample infected with SS2, while fewer dead cells were found in the morin-treated samples (**Figures 2A,B**). The release of LDH by J774 was assessed to quantify such protection. In agreement with the above results, morin treatment significantly alleviated SS2-mediated cell injury in a concentration-dependent manner (**Figure 2B**). Additionally, almost no cell injury was observed in the cells treated with only morin, suggesting that this compound had no cytotoxicity in J774 cells (**Figure 2A**).

Suilysin has been proven to play a crucial role in the process of SS2 traversal across the epithelial barrier (Du et al., 2013; Leung et al., 2014). To determine the effect of morin on the penetrability of pathogenic SS2, J774 monolayers were used to model the epithelial barrier by plating into transwell permeable supports, as described above. We found that morin-treated SS2 failed to pass though monolayer cells while the control group allowed for pathogenic traversal (**Figure 2C**), indicating that morin inhibits SLY function in a similar manner to cholesterol, which is consistent with previous findings (Charland et al., 2000). Taken together, our results establish that treatment with morin significantly inhibits the penetration of SS2 through the epithelial barrier and SS2-induced cell injury.

### Morin Protects Mice from SS2 Infection

**FIGURE 3:**
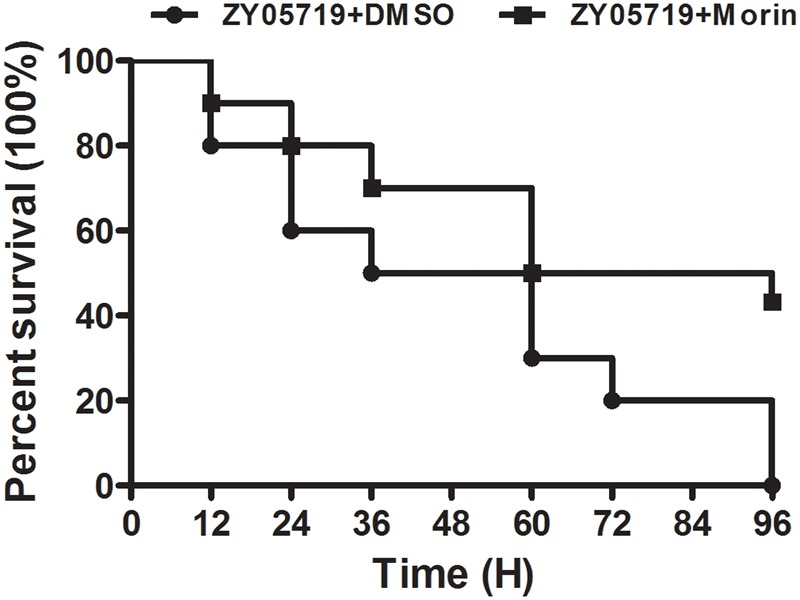
**Morin treatment significantly inhibits the mortality of infected mice.** Infected mice were treated with morin or DMSO as a negative control, and the mortality of infected mice was statistically analyzed for 96 h using the Kaplan–Meier method.

To examine whether the protection of morin against *S. suis* virulence was also observed in vivo, the survival rate of SS2-infected mice treated with morin was compared to untreated infected mice. Importantly, the survival rate of infected mice was significantly different between the morin-treated and untreated groups. Consistent with previous findings, all infected mice were dead at 96 h post-infection (**Figure 3**); however, this mortality rate was decreased to 56.67% (**Figure 3**). In summary, our results suggest that morin treatment leads to a substantial inhibition on SS2-induced death in infected mice.

### Determination of the Molecular Mechanism Underlying Morin Inhibition of SLY Activity

**FIGURE 4:**
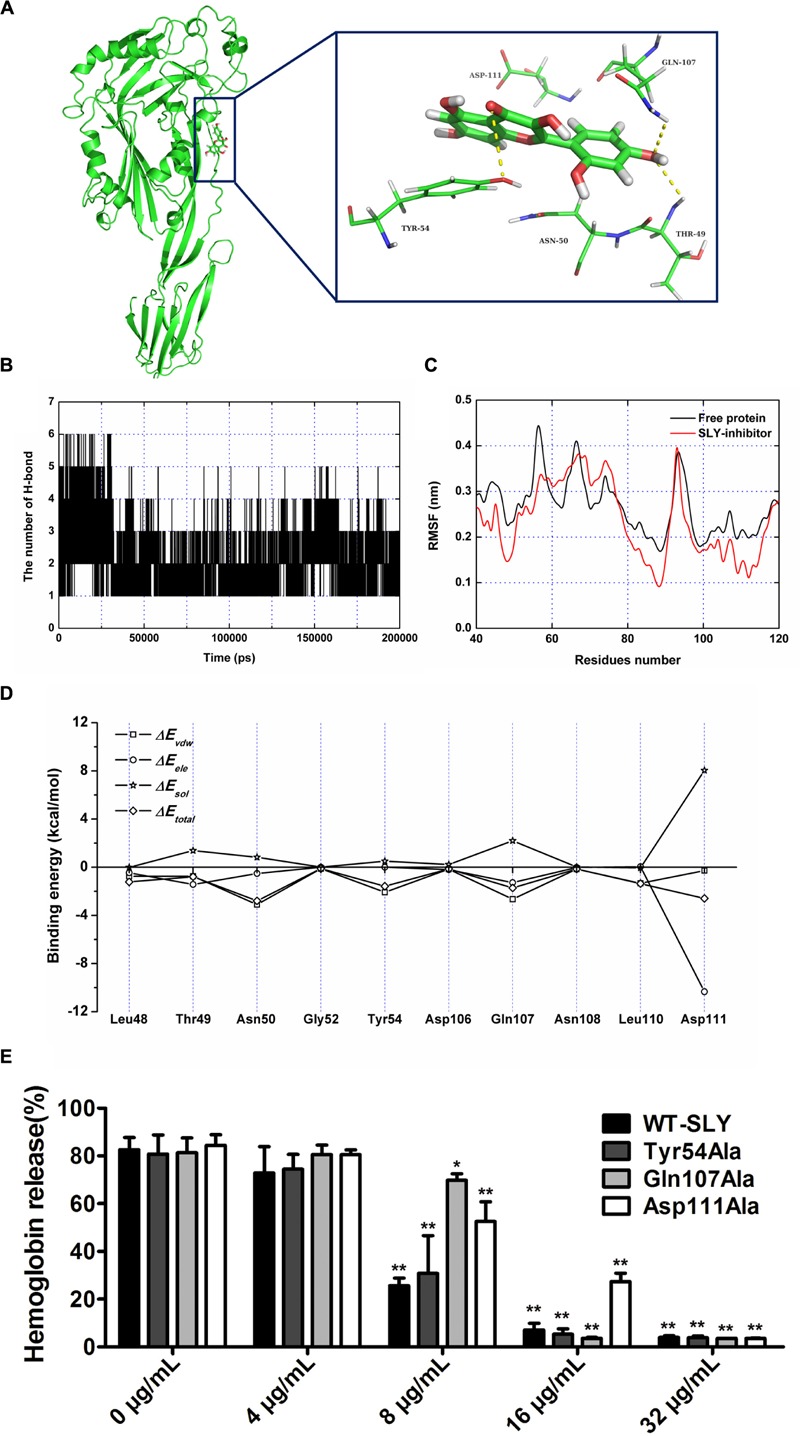
**The binding mode of morin with SLY based on the MD simulation. (A)** The 3D structure and residues of the binding site in the SLY-morin complex; **(B)** RMSF of the residue positions over the 200-ns simulations with respect to their initial position in SLY as a free protein and in complex; **(C)** The number of hydrogen bonds between morin and SLY during the MD simulation; **(D)** Decomposition of the binding energy on a per-residue basis in the binding sites of the SLY-morin complex; **(E)** The effect of morin on the hemolylsis induced by SLY and its derivatives, Tyr54Ala, Gln107Ala, and Asp111. ^∗^ indicates *P* < 0.05, and ^∗∗^ indicates *P* < 0.01 compared with the morin-free sample (two-tailed Student’s *t*-test).

Over the 100-ns of MD simulation, it was found that morin localized to domain 2 of SLY, as shown in **Figure 4A**. From the binding mode of SLY-morin, it was shown that there are three strong hydrogen bonds between morin and Thr49, Tyr54, and Gln107. Moreover, Asn50 and Asp111 also have a strong hydrophobic interaction with morin (**Figure 4A**). The number of H-bonds between morin and SLY during the 200-ns simulation are plotted in **Figure 4B**, which is consistent with the above results.

It is widely accepted that the flexibility of the residue can be revealed by the calculation of the root mean square fluctuation (RMSF) of the protein. As such, the RMSF of these residues around the binding sites of the SLY complex and free SLY are shown in **Figure 4C**. Interestingly, our analysis reveals that the flexibilities in the SLY binding sites are different in the presence and absence of morin, which suggests that the residues in the binding sites of the SLY complex are more rigid as a result of ligand binding. In **Figure 4C**, the residues (50–60 and 100–120) in the protein-morin binding sites show a small degree of flexibility with RMSF of less than 0.1 nm when compared with free protein, indicating that these residues seem to be more rigid as a result of ligand binding.

To obtain detailed information of the residues in the SLY-morin binding region and their contribution to the whole system, the ligand-residue interaction decomposition was calculated for the complex system by an MM-PBSA method. As shown in **Figure 4D**, Thr49, Gln107, and Asp111 provide appreciable electrostatic contributions, with ΔEele values of ∼–1.43, ∼–1.28, and ∼–10.34 kcal/mol, respectively. Moreover, Asn50, Tyr54, and Gln107 have the strongest attraction interaction, with van der Waals values of ∼–3.10, ∼–2.08, and ∼–2.65 kcal/mol, respectively. With the exception of Asp111, the majority of the decomposed energy interactions originated from Van der Waals interactions, while electrostatic contributions appeared to be a minor influence on these key residues. Based on the above results, we conclude that the amino acid residues of Thr49, Asn50, Tyr54, Gln107, and Asp111 may be important for morin binding. Hemolysis assay was further performed to detect the inhibitory activity of morin on hemolysis induced by rSLY and its derivatives. Three SLY mutants (Tyr54Ala, Gln107Ala, and Asp111Ala) remain active for lying defibrinated rabbit blood (**Figure 4E**), however, the inhibition of morin on these mutations was alleviative than WT-SLY (**Figure 4E**).

To confirm this hypothesis, the total binding free energy for the SLY-morin complex and their detailed energy contributions (calculated according to the MM-PBSA approach) are summarized in **Table 1**. According to the theoretical results, the binding free energy (ΔGbind) of the interaction between morin and protein is greater for WT-SLY than mutant SLY, indicating that WT-SLY has the strongest ability to bind with morin. By fluorescence spectroscopy quenching, we measured the binding constants (KA) and the number of binding sites between morin and the three mutants, and the results of this analysis were highly consistent with those obtained by computational biology methods (**Table 1**). These findings indicate that the information generated by the molecular modeling of the SLY-morin complex is reliable.

**FIGURE 5:**
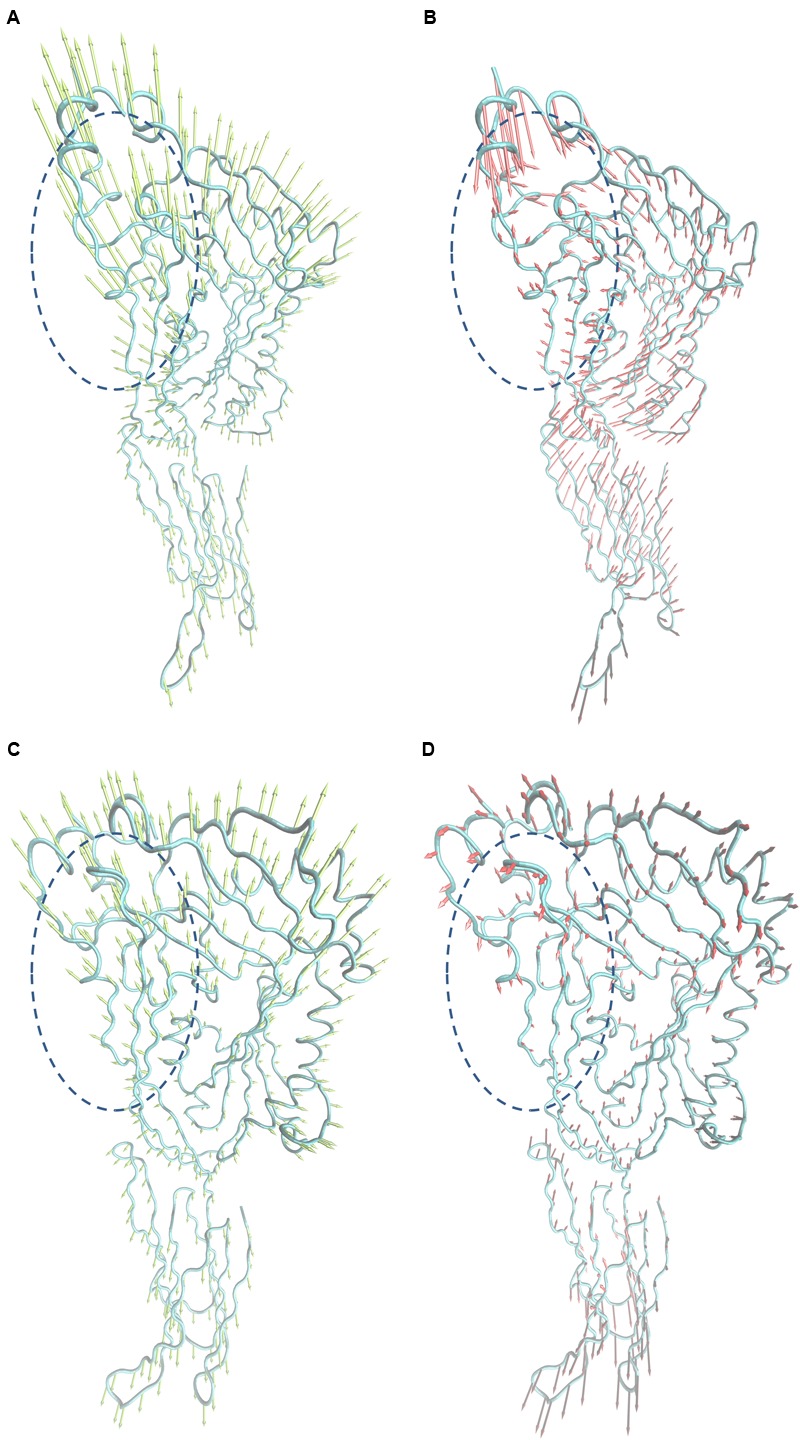
**Collective motions obtained by principal component analysis on the simulation trajectory. (A)** and **(B)** Motions corresponding to PC1 and PC2 of the free SLY. **(C,D)** Motions corresponding to PC1 and PC2 of the SLY-morin complex.

**FIGURE 6:**
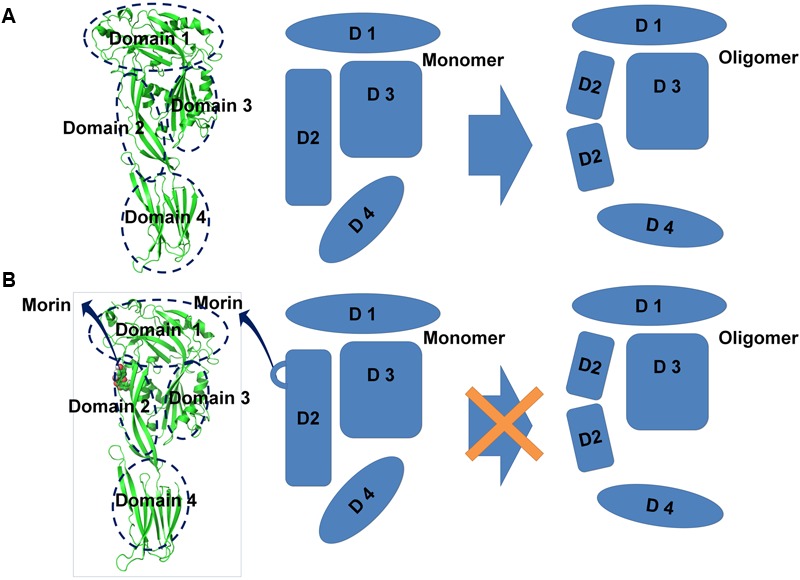
**Illustration depicting the mechanism underlying morin-mediated SLY inhibition. (A)** During the transition from monomer to oligomer, many aspects of the conformational changes have been directly observed, including the buckling of domain 2; **(B)** Binding to morin blocks the conformational change of domain 2 from the monomeric to the oligomeric form, leading to the loss SLY lytic activity.

To explore the mechanism of SLY inhibition by morin, a principal component analysis (PCA) was performed on the MD trajectory of free SLY and the SLY-morin complex to identify the most significant motions of the protein. The first component (PC1) of free SLY is composed of several motions (**Figure 5A**): as a rigid body, there is an extended motion to the entire conformation of the protein, particularly to the “stem” domain 2 (represented by the dotted line in **Figure 5A**). This motion is sufficiently large to meet (but not exceed) the requirements for the conformational transition of SLY from its monomer form to an oligomer. The second principal component (PC2) primarily corresponds to the slight vibration of the protein backbone, as shown in **Figure 5B**. The PC1 of the complex is composed of several motions, similar to that of the free protein, with the exception of the motion in domain 2 (represented by the dotted line in **Figure 5C**). The second principal component (PC2) was shown in **Figure 5D**. As shown in **Figure 5C**, the motion of domain 2 is clearly weaker than that of free SLY. It should be noted that domain 2 is also the binding site of morin, based on previous data. Therefore, it is confirmed that the motion of domain 2 is restricted by the binding of morin. According to previous reports, the toxin protein of the highly conserved CDC family can form the largest transmembrane β barrels. During pore assembly, many aspects of pore formation have been directly observed, including the buckling of domain 2. In this work, the conformational change of domain 2 was clearly blocked upon morin binding, inhibiting the formation of oligomers (**Figure 6**) and causing the hemolytic activity of SLY to be lost.

## Discussion

To date, the ongoing exploration of antibiotics lags behind the apparent ceaseless emergence of antibiotic-resistant bacteria (Escaich, 2008). The irrational overuse of antibiotics in humans and animals drives microbial mutagenesis and subsequent resistance (Marra, 2006). Thus, the development of novel strategies to fight antibiotic resistance and bacterial infection has gradually evolved into an inevitable demand. The targeting of virulence factors is an innovative strategy that exerts no selective pressure on pathogen survival but instead lessens bacterial pathogenicity (Dobrindt and Hacker, 2008; Rasko and Sperandio, 2010). Because SLY is important for the virulence of SS2, the suppression of SLY is a potential approach that may decrease SS2 virulence and reduce infectious symptoms without affecting bacterial viability.

In this study, morin was identified to be an efficient inhibitor of SLY by attenuating the hemolytic activity of SLY and was verified to have no observed antibacterial activity against SS2. Although the negligible antibacterial activity of morin allows bacteria to reproduce in an unrestrained setting, this compound can limit SS2 pathogenicity. In our in vitro co-culture system, morin treatment significantly alleviated SS2-induced cell injury and epithelial barrier penetration according to a cytotoxicity assay and transwell experiment in cell models. In vivo experiments showed that treatment with morin also reduced death rates in C57BL/6J mice infected with the aggressive SS2. The effect of morin on SS2 virulence may be related to its anti-SLY activity. Thus, the inhibition of SLY activity by morin decreases the severity of infections caused by SS2 strains.

In this work, a standard molecular dynamics simulation for the SLY-morin complex was performed. On the basis of the MD simulation and the binding free energy calculations, we found that morin could bind to the domain 2 of SLY by forming strong contacts with residues Thr49, Tyr54, Gln107, Asn50 and Asp111. These results were confirmed by ligand-residue interaction decomposition using the MM-PBSA method, residue point mutations, and a fluorescence-quenching assay. To further explore the mechanism underlying SLY inhibition by morin, a principal component analysis (PCA) was performed on the MD trajectory of the free SLY and SLY-morin complex to identify the most significant motions of the protein. Based on our analysis of dynamic trajectory, we can predict that the motion of domain 2 is restricted by the binding of morin which blocks the conformational change of domain 2 from the monomeric to oligomeric form to inhibit the lytic activity of SLY. In addition, the declined sensibility of morin on hemolysis of SLY mutations futher indicated that Tyr54, Gln107, and Asp111 are three important amino acid residues for the engagement of morin with SLY.

Morin is a flavonoid constituent extracted from Chinese herbs and was reported to exhibit many biotic activities, such as anti-inflammation and inhibition of the catalyzation of *S. aureus* SrtA (Fang et al., 2005; Kang et al., 2006) In our previous study, morin treatment reduced the hemolytic activity of *S. aureus* α-hemolysin (Wang et al., 2015). LLO and SLY both belong to the cholesterol-dependent cytolysin family (Heuck et al., 2010), suggesting that morin may inhibit the activity of all CDC family members and other Gram positive bacteria that secrete pore-forming toxins. In summary, morin represents an alternative for the treatment of SS2 infections. Further research into morin may provide a new therapeutic approach for antibiotic-resistant Gram positive bacterial diseases.

## Ethics Statement

Eight-week-old female C57BL/6J mice were purchased from the Experimental Animal Center of Jilin University (Changchun, China). The animal experiments were reviewed and approved by the Animal Care and Use Committee of Jilin University.

## Author Contributions

XD and JW conceived and designed the experiments. GL, GjL, ZQ, HL, LW, YW, BL, and XN performed the experiments. JW and XD contributed reagents/materials/analysis tools. JW and XD wrote the paper.

## Conflict of Interest Statement

The authors declare that the research was conducted in the absence of any commercial or financial relationships that could be construed as a potential conflict of interest.
